# Supplementary figures and images for: The child’s pantheon: Children’s hierarchical belief structure in real and non-real figures
Source: PLoS One. 2020 Jun 17;15(6):e0234142. doi: 10.1371/journal.pone.0234142 (PMC7299553; doi:10.1371/journal.pone.0234142)

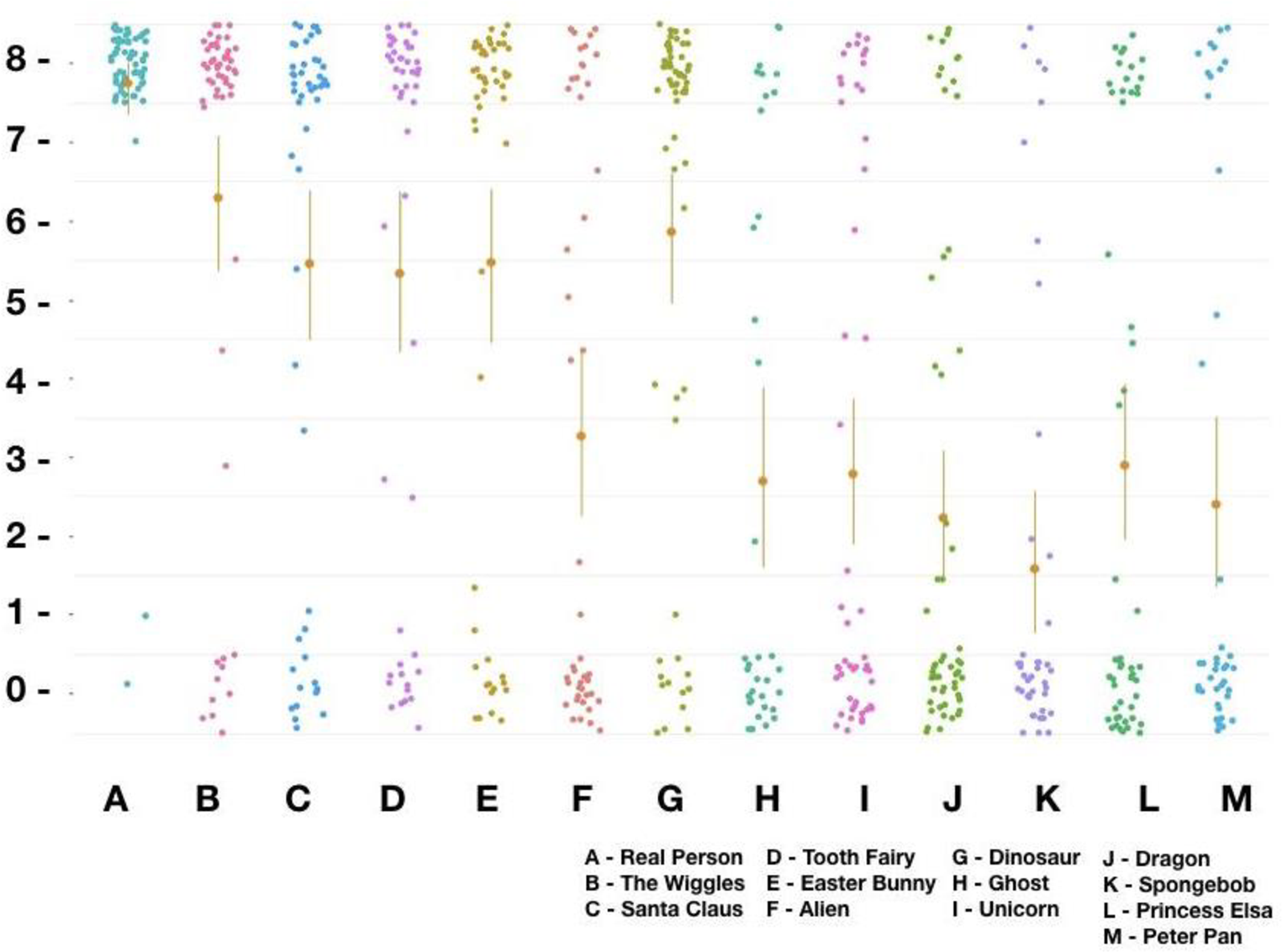

Supplement: S1 Fig — (TIF) [file pone.0234142.s001.tif]

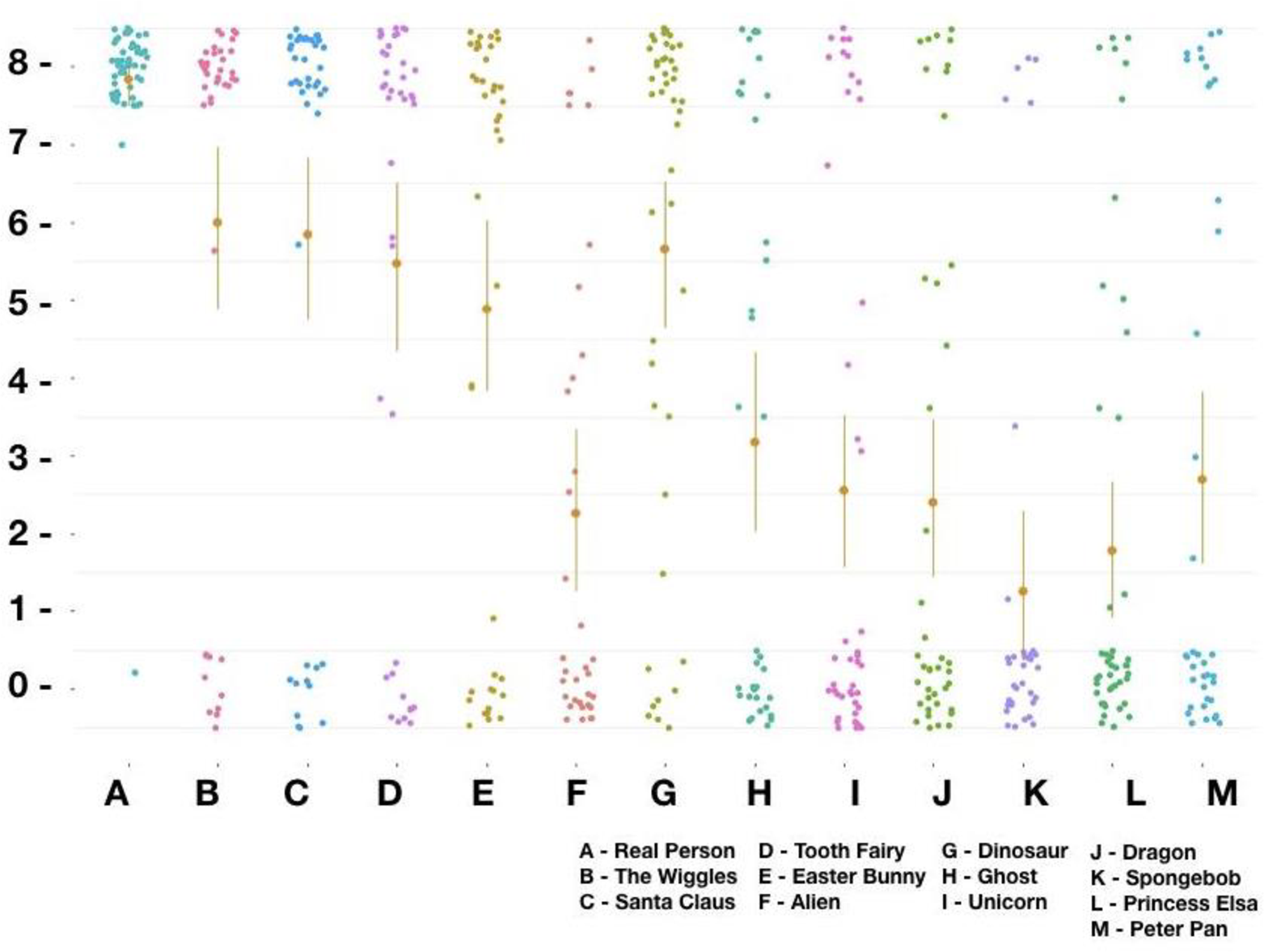

Supplement: S2 Fig — (TIF) [file pone.0234142.s002.tif]

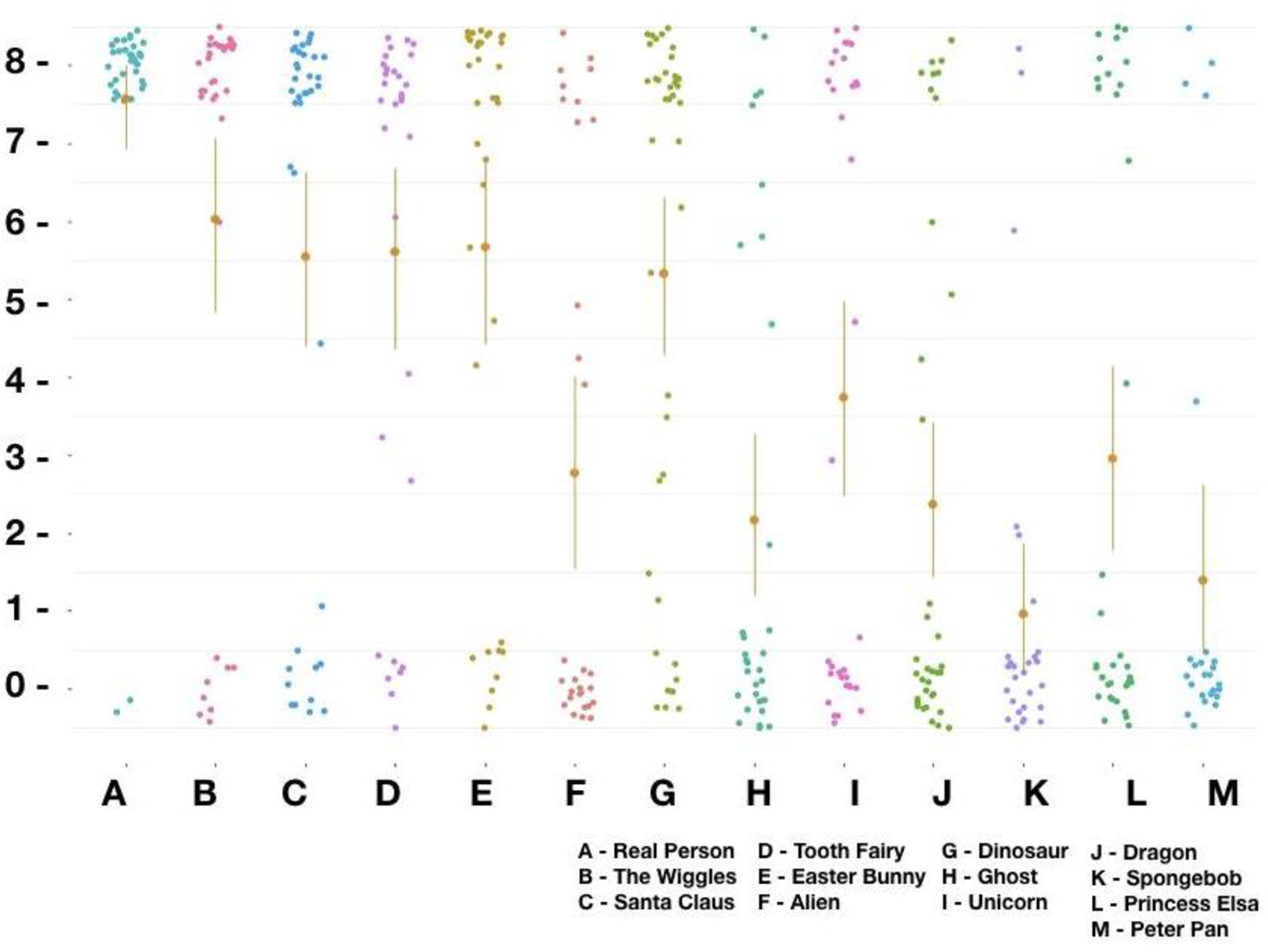

Supplement: S3 Fig — (TIF) [file pone.0234142.s003.tif]

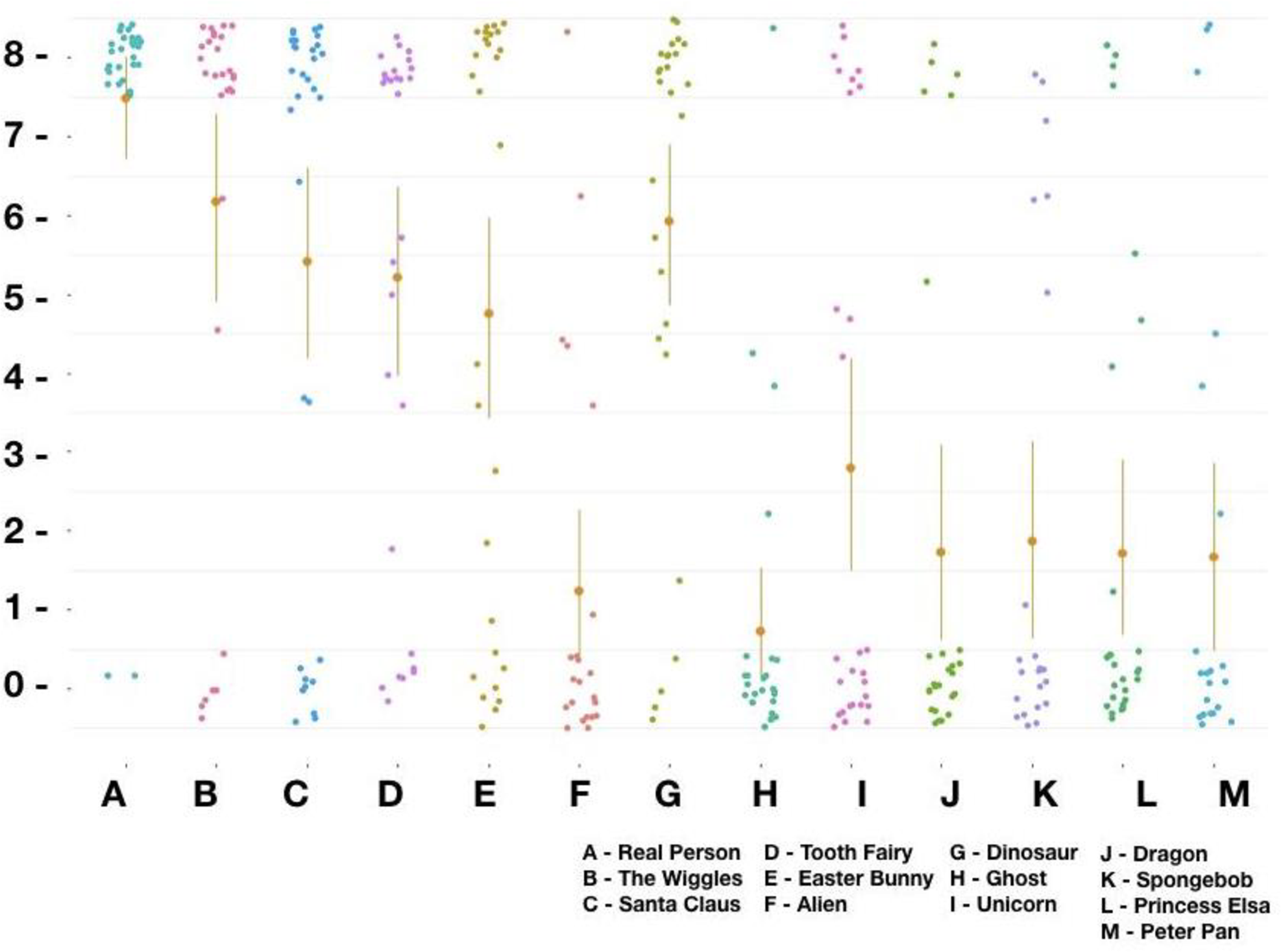

Supplement: S4 Fig — (TIF) [file pone.0234142.s004.tif]

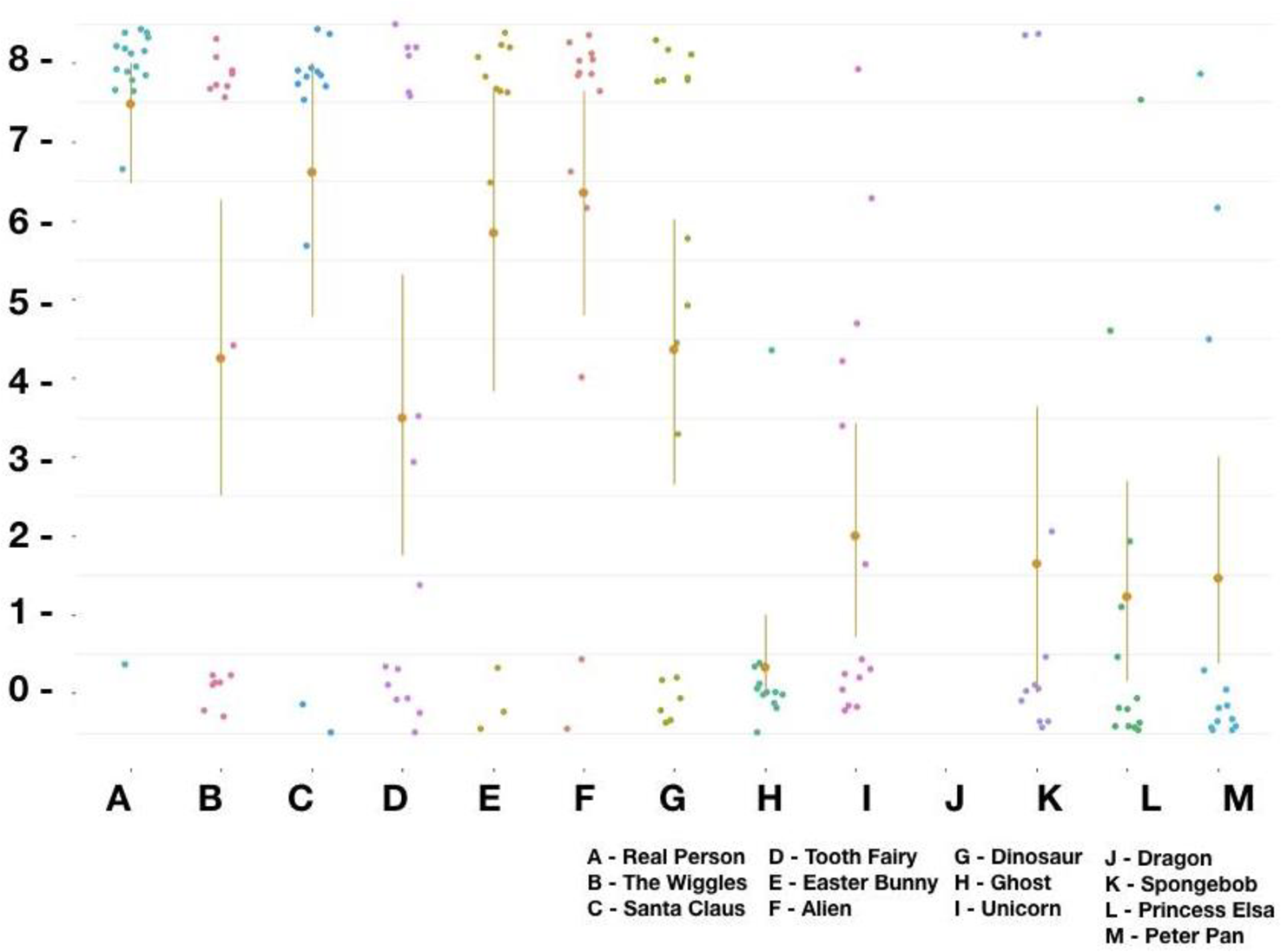

Supplement: S5 Fig — (TIF) [file pone.0234142.s005.tif]

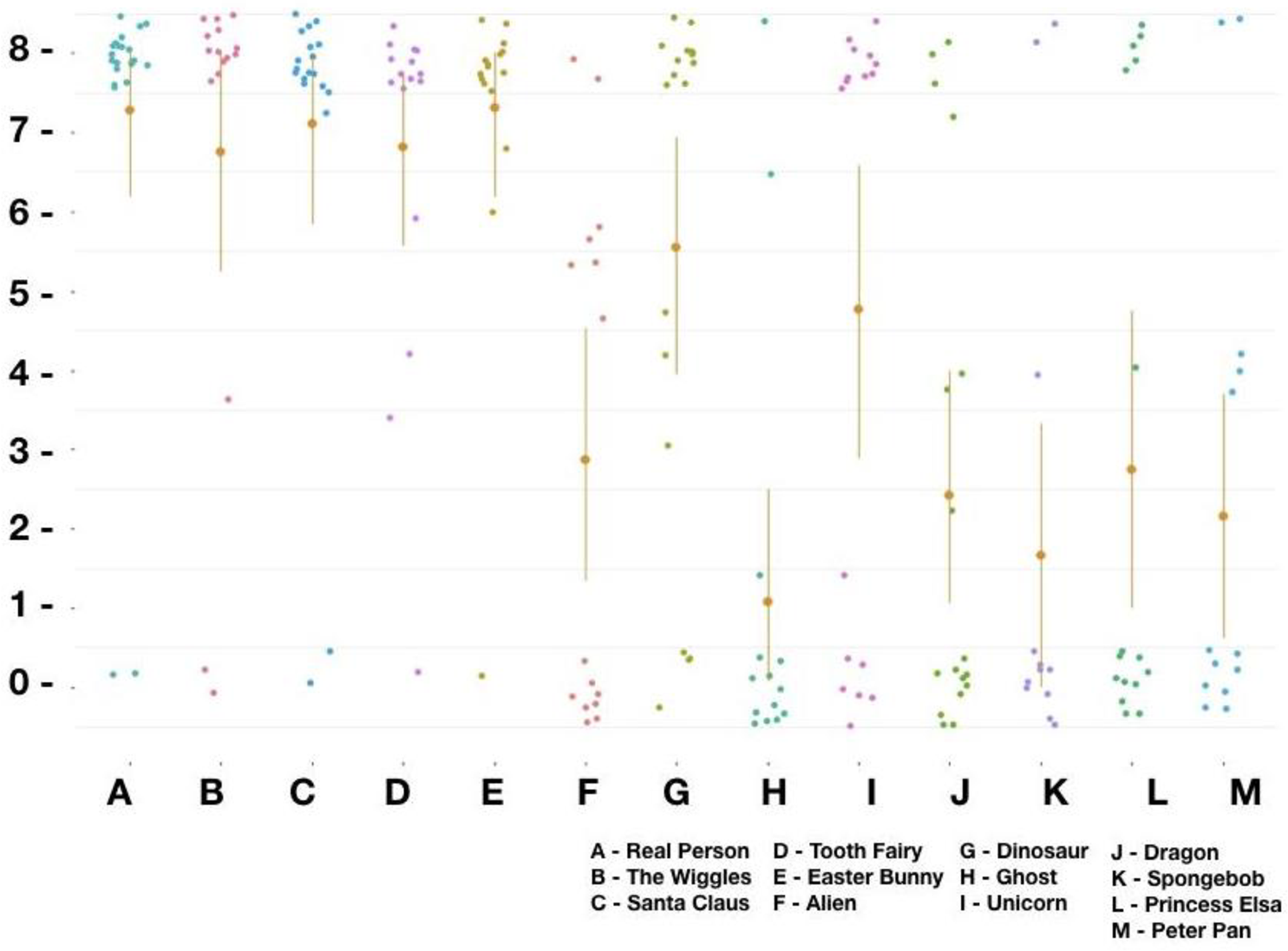

Supplement: S6 Fig — (TIF) [file pone.0234142.s006.tif]
