# Supplementary material for: The child’s pantheon: Children’s hierarchical belief structure in real and non-real figures
Source: PLoS One. 2020 Jun 17;15(6):e0234142. doi: 10.1371/journal.pone.0234142 (PMC7299553; doi:10.1371/journal.pone.0234142)
Supplement: S1 Appendix — (DOCX) [file pone.0234142.s007.docx]

**Appendix S1.**

Parents agreed to the following conditions before helping their child complete the survey.

*In conducting this kind of research, it's very important that all children who answer these questions answer them under conditions that are the same. For that reason, we would hugely appreciate you following these simple instructions.*

1. *Please do not correct or guide your child to a particular answer, or correct your child if they provide a 'wrong' answer.*
2. *Please read text to your child as it is written. Sometimes you may need to change some of the words for the benefit of your child, so please only alter the delivery consistent with the spirit of the question. Please be careful to observe point 1.*
3. *If your child asks you questions, please answer them as best you can without guiding your child to a particular response. If this seems impossible, please say to your child "Good question, how about I answer that question after we're finished playing this game - why don't you try to answer the question now, ok?"*
