# Supplementary material for: The child’s pantheon: Children’s hierarchical belief structure in real and non-real figures
Source: PLoS One. 2020 Jun 17;15(6):e0234142. doi: 10.1371/journal.pone.0234142 (PMC7299553; doi:10.1371/journal.pone.0234142)
Supplement: S1 Data — (DOCX) [file pone.0234142.s008.docx]

**Supplementary material A.**

**A note on data collection and analyses.**

Data collection for this experiment was executed over the course of 12 months in a longitudinal manner, and several additional hypotheses were proposed for this larger within-participants dataset. However, due to attrition we were not able to perform these planned analyses. In the body of the manuscript we have included analyses for hypotheses based on the data from the first wave of data collection. Here we will describe how data were collected over the course of the year, and present the values as appropriate. Our full datasets are available online at <https://osf.io/wurxy/>, and we urge interested readers to access this data.

We also intended to ask children were asked a number of epistemological questions regarding each figure. These questions were collected primarily to inform longitudinal data, which would have been considerably more highly powered. Moreover, due to the number of target figures, and the fatigue such questions can impose on children, we asked these questions of only a limited number of targets at each timepoint. Ultimately we asked children about each target figure twice, separated by roughly six months. However, due to attrition this data is not particularly informative. Hereafter, we will present summary statistics from the first wave of data collection, and direct motivated readers to our publically available data if they wish to view the rest of the data (available on the <https://osf.io/wurxy/>).

**Recruitment Procedures and Participants**

Data recruitment and collection was the same at each subsequent time point as was described in the body of the manuscript. After the first wave of data collection, subsequent data collection were conducted on average every 52 days. In total, we collected data a total of 7 times. Each administration of the study was as described in the body of the manuscript.

**Data Collection**

After the first wave of data collection, we recognized that missing data on figures might be the participant using ‘0’ to indicate complete lack of belief, or may simply represent missing data. Thus, after the first time point, children were asked to confirm (using a binary scale) their disbelief, so that we could determine whether a non-response on the focal question was a response of zero-endorsement, or simply missing data.

**Longitudinal Hypotheses**

In addition to the ‘static’ hypotheses described in the body of the manuscript, we made several hypotheses regarding the longitudinal data.

**H5:** We will conduct longitudinal analysis on category of figures to determine whether or not children’s belief becomes more adult-like (i.e., declines) over the course of one year’s development.

**H6:** We will conduct longitudinal analysis on individual cultural figures (i.e., Santa, Easter Bunny, and Tooth Fairy) to examine whether or not time of year (i.e., corresponding to specific ritual events, such as Christmas and Easter) influences belief.

**H7:** Contingent upon a significant and positive increase at belief associated with time of year, we intended to explore whether or not there was a halo effect associated with time of year, such that belief in other figures increased during Christmas or Easter (i.e., is christmas really ‘a magical time of year’).

**Data Treatment**

At T1 data were collected on 9-point likert scale (presented in the form of gold stars for children). However, after our first round we identified that a missing value on this scale may be a missing value, or an indicator of lack of endorsement. In wave T2 through T7 we used an 8-point scale, and employed a radio-button that participants could select to confirm that ‘*[They] did not think [x] was real*’ (if present, participants were scored a ‘0’ on the likert scale, thus making it a 9-point scale in total). Data in T1 were converted such that their original score was reduced by 1 so that scores across all waves of data collection were consistent.

**Results**

**Epistemological Beliefs**

In the following S1A-S1D Tables we present the absolute and proportional values of responses for children’s answers to epistemological questions collected during the first wave of data collection.
