# Supplementary material for: The child’s pantheon: Children’s hierarchical belief structure in real and non-real figures
Source: PLoS One. 2020 Jun 17;15(6):e0234142. doi: 10.1371/journal.pone.0234142 (PMC7299553; doi:10.1371/journal.pone.0234142)
Supplement: S2 Data — (DOCX) [file pone.0234142.s009.docx]

**Supplementary material B. Training questions**

The following are the training questions, presented in order, for the primary study:

1. Are chairs real?
2. Are chairs that sing real?
3. Are elephants with long noses real?
4. Are elephants with short noses real?
5. Are rocks that float in water real?
6. Are trees that grow upside down real?

The following are the training question, presented in order, for the replication study. Each answer was re-inforced with the child. For example, if they selected 10/10, or 1/10, the experimenter (without correction or judgement) clarified that the child had endorsed, or denied, the existence of the thing in question. For the Blergo, scores greater than 1 were reinforced with the clarification that “*Ok, remember a 'Blergo' was something I just made up in my head. There are no right or wrong questions, just remember that if you say a number bigger than 1, you're saying that it is sort of real. “*

[read to the child] *The next question I will ask you is how 'real' do you think the penguin is. If you think it's really real, as a real as your mum or your dad, you should make all 10 stars yellow. If you think that a Penguin is not real, that's ok. If you think Penguins are not real at all, you should select only 1 star. If you're not sure, then you should select a different amount of stars. If you think it's probably real, then choose a big number of stars, if you think it's probably not real you should choose a smaller number of stars.*

Do you think that Penguins are real?

[read to child] *Ok, I'm going to ask you about a thing I just made up. It doesn't really exist. It's just an idea that is in my head. Do you think that a 'Blergo' is real? Remember: If you think a Blergo is really real, as a real as your mum or your dad, you should make all 10 stars yellow. If you think that a Blergo is not real, that's ok. If you think a Blergo is totally fake, you should select only 1 star. If you're not sure, then you should select a different amount of stars. If you think it's probably real, then choose a big number of stars, but if you think it's probably fake you should choose a smaller number of stars.*

Do you think that a 'Blergo' is real?

[read to child] *I want you to think of an Elephant now. But I want you to think of a special Elephant, one that has a short nose. Remember, 10/10 means that it's really real, 1/10 means it's not real at all, and numbers between 1 and 10 are in the middle! Do you think that Elephants with short noses are real?*

Do you think that Elephants with short noses are real?
