# Supplementary material for: The child’s pantheon: Children’s hierarchical belief structure in real and non-real figures
Source: PLoS One. 2020 Jun 17;15(6):e0234142. doi: 10.1371/journal.pone.0234142 (PMC7299553; doi:10.1371/journal.pone.0234142)
Supplement: S1 Table — (DOCX) [file pone.0234142.s010.docx]

S1A-S1D .Tables. Responses to epistemological question for four figures.

**
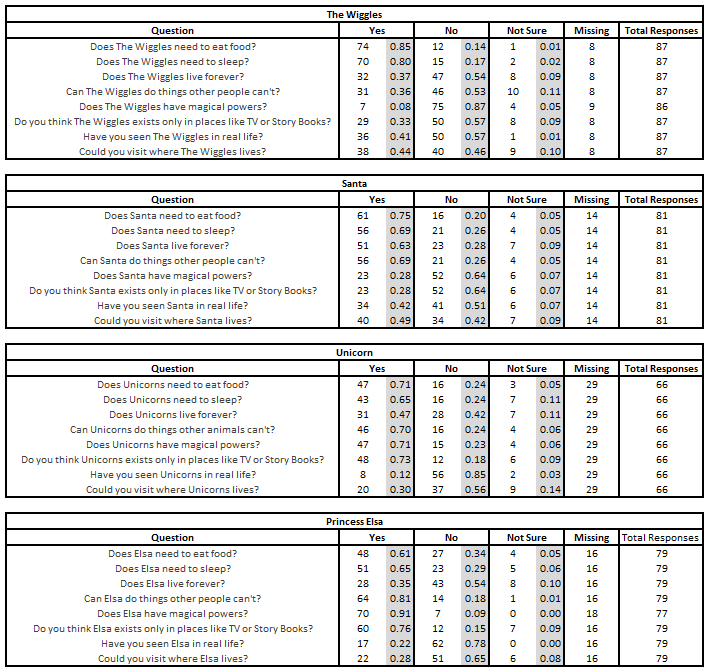
**

**Children’s Belief Longitudinally**

Our hypotheses involved conducting longitudinal analyses in order to to determine whether or not children’s belief becomes more adult-like (i.e., declines) over the course of one year’s development. Our sixth and seventh hypotheses were our most novel, and proposed that belief for individual cultural figures (i.e., Santa, Easter Bunny, and Tooth Fairy) may have fluctuated with the time of year (i.e., with figure-specific corresponding ritual events). We had hoped to identify if Christmas and Easter, respectively, would temporarily increase belief for Santa and the Easter Bunny. We were unsure, a) if this would occur at all, and b) if it did occur, would it occur before, during, or after the event (corresponding with different levels of engagement and testimony). The tooth fairy was to be included as a control condition, as visitation by the tooth fairy are uniformly distributed through the year. Further, we wished to explore whether or not large culturally ritualistic events had a kind of halo effect for other figures - was christmas really a ‘magical time of year’, so to speak, and would it increase belief in other figures.

Unfortunately, we noted considerable attrition, such that of the 95 children who participated at T1, only seven individuals participated in all seven timepoints, 12 participated in 6 or more, 22 participated in five or more, and 44 participated in four or more. Moreover, of this subset, not all children answered all questions at each timepoint. While it would be possible to impute values, it would corrupt the integrity of the analyses to such an extent that any interpretation would be meaningless. Thus, we do not present our intended longitudinal analyses here. However, we have also included plots for each time point. More importantly, our data (for each time point) is publicly available at <https://osf.io/wurxy/>, as is our R code (thus making all figures and value reproducible).
