# Supplementary material for: The child’s pantheon: Children’s hierarchical belief structure in real and non-real figures
Source: PLoS One. 2020 Jun 17;15(6):e0234142. doi: 10.1371/journal.pone.0234142 (PMC7299553; doi:10.1371/journal.pone.0234142)
Supplement: Attachment — Submitted filename: Response to Reviewers.docx [file pone.0234142.s011.docx]

**Review Comments to the Author**

Reviewer #1: This is an interesting and thoughtful attempt to examine children's ontological beliefs in a more nuanced fashion than is customary. To simplify somewhat, a great deal of past research has assumed that children classify entities in a dichotomous fashion - as real or unreal. Instead, the authors argue, children have a more differentiated notion of reality with real people at one end, fictional characters at the other, with other categories in between.

Despite the strength of the theoretical framing I have several reservations about the data that the authors offer in support of their thesis.

1. In both studies, the authors asked a wide age range of children to situate each character on a 9-point star scale using training feedback that included the following: " If you think it's probably real, then choose a big number of stars, if you think it's probably not real you should choose a smaller number of stars". I think the assumption that children, especially preschool children, can understand varying degrees of probability and translate them into a varying number of stars is quite dubious. The fact that similar results were obtained whether or not parents served as the interviewer does not show that children understood the scale. For all we know, children were subject to the same misunderstanding or partial understanding of the response scale no matter who interviewed them.

The reviewer suggests that the reliability (via replication) does not translate into true understanding (on the part of the children). We agree, but the purpose of the replication was not to demonstrate that children understood the scale, but rather to demonstrate that these tasks could indeed be conducted at home, with parents as interviewers, with reliable results. This reviewer’s argument implies that, if it is the case children can’t make probabilistic claims about reality, then we must accept that the status quo measurements of ‘true’, ‘untrue’, and ‘not sure’ are, in fact, the most valid way to measure belief (that is, only categories, and not probabilities are appropriate). (We do, however, suspect this claim is untrue after a certain point: say, 7 or 8 years. Children are quite sophisticated at this point, as I’m sure the reviewer is aware. If this is the case, younger children should be fairly variable (due to mis-comprehension), or that their bias reliably skews in one direction (as evinced by the replication; putting aside the possibility of a category-misunderstanding interaction). And so we must ask: does mis-understanding among young children -- across the sample and the figures -- lead to an over- or under- estimate of endorsement (assuming such a thing could be accurately measured). If there is more bias, or more variance, the most misleading scenario for our data is that we describe a bias towards gullibility. If so, then 4, 5, & 6 year olds - in reality - are cannier in their understanding of reality-fantasy than we researchers can appreciate (due to a ‘gullibility bias’ in reporting). If that is the case, then younger children in particular are more skeptical (in reality) about both unreal figures, but also about real figures. The positive slope associated with ‘real person’ (figure 4, 9a) suggests this claim is partially true (assuming young children recognize that real people are, in fact, real). However, the replication shows a flat line for the real figure, and both datasets veer towards bimodal distributions (Figure 7). We interpret the middling values as nuance, but it’s also possible it’s misunderstanding. That said, does the reviewer believe that scores at the extremes are the product of misunderstanding, too? Such that children were unsure what 1, or 9, on the scale meant (even after training suggests that they do understand how to use scales; see page 13/14)? If it is the case that children are not capable of probabilistic endorsement, then perhaps a more appropriate conception of reliable mis-understanding/bias is a push away from the mid-values, and towards the extremes. If this is so, then we’d expect bimodal data… which is largely what we report (noting that we met assumption checks prior to conducting these analyses). If this is the case, then the use of linear models using the means of bimodal data are, in fact, highly conservative, and we are under-reporting the relationships between age and endorsement.

We’re not trying to be clever here, only to say that while it is likely the case that individual children’s capacity to comprehend the question is partially true (indeed, the meaningfulness of error term for all of social and developmental psychology is widely known, if not widely discussed in the literature), the replication gives us confidence that at least it’s reliable. We do not believe that our data -- particularly the data with older children -- are so corrupted by measurement error that these analyses and results are uninformative. Indeed, in the absence of any other scholars attempting such a quantification, we must conclude that this is a very hard problem to solve, and hope that our results are useful despite certainly containing weaknesses upon which others will improve. Given these limitations, we have emphasised the following caveats in the manuscript to ensure readers are not under the impression that we are suggesting these are water-tight results, but that these are first results, which are replicable, and that scholarly work may progress from that point. We highlight that we dedicate 1200 words to this very topic in the discussion, beginning at the paragraph: *We believe there are three primary limitations...*

2. Eyeballing the data in Figure 5 (Endorsement for categories), there seems to be little justification for claiming that children differentiate between virtually real characters (e.g. The Wiggles) and cultural figures (e.g. Santa Claus) or between Ambiguous figures (e.g. ghosts) and Fictional Figures (e.g. unicorns). A more cautious and defensible conclusion might be that children make a 3-way split between real figures, less real figures and doubtful figures.

We are sympathetic to this claim, and have spent time attempting to to understand what would convince us, as authors, of the utility of our [own] claim. We note with no disrespect that the reviewer is relying on their intuition that similar means imply similarity between measures, which should serve as the basis for groupings (in this case, figures into categories). We went to great pains to avoid this, by conducting the cluster analysis: we examined the variation and co-variation in all scores based on the input of the children. We fully acknowledge that a suitable solution using this analyses can include ALL the figures into one super-ordinate category. But we predicted certain categories, and transparently report when they conformed, and when they did not. The author argues that a ‘more cautious and defensible conclusion’ might involve more clumping than splitting, which is certainly a position one might argue for. We do not make this distinction the basis for our work (afterall, we had very fine-grained predictions, and so would like to report fine-graned results). We have, however, included the following in our discussion on this matter (see page 33):

*During the peer-review process, a reviewer suggested that rather than the multiple categories we propose, it may be more sensible - based on an interpretation of mean values for category data (see Figure 8) - to suggest only three categories: real, less real, and doubtful. We are not opposed to a ‘clumping’ strategy over a ‘splitting’ when making future hypotheses about this proposed pantheon. The question all researchers ought to ask is: what utility do my distinctions serve? In the present case, we had rather fine-grained hypotheses about the structure of the pantheon, and wished to report and interpret at a corresponding level. Certainly three, rather than five, categories may serve some purposes particularly well, though we hope that our data - imperfect that it is - can serve as a foundation for future theory-building based on the relative contribution of different forms of evidence and testimony, as described in brief by Woolley and Ghossainy (2013). Future research may examine the nature of these differences and similarities using alternative statistical and theoretical models, though we again note that our data is publicly available for the interested reader, and we are open to future discussion and collaboration on this topic. We maintain, however, that our results, measurement, and analyses suggest that children are generally capable of admitting nuance and ambiguity into their reality endorsements, and this is apparent at population levels. That said, that we replicated our results in a second study using traditional methods of enquiry should provide some confidence that the question is reliable and face-valid.*

3. The authors elaborate on various ways in which different categories of figure might be distinguished (see Table 2 for a listing of these criteria). However, they provide no evidence that these criteria were deployed by children in making their reality judgements. In particular, the authors did not ask children to offer a justification for their judgments even though such justifications might have lent support to the authors' claims. In the absence of such justification data, the authors have to rely on the reality judgment data alone and yet there are all sorts of reasons why children might make different reality judgments for different figures. For example, Figure 3 suggests that children were less confident of the existence of Peter Pan than the Tooth Fairy. This might reflect the fact that children receive indirect evidence for the Tooth Fairy and not for Peter Pan (as implied by the criteria set out in Table 2) but another equally plausible possibility is that children hear more references to the Tooth Fairy (and from more people) as compared to references to Peter Pan.

We are sensitive to the claim that we don’t show direct evidence for the role of ritual (or anything else) in our paper. The reviewer also suggests that the prominence of some figures (like the tooth fairy) would lead to greater endorsement over other figures (like Peter Pan). We will return to this point momentarily.

What we hoped to provide here is a novel and relatively thorough literature review on an established topic using an expanded theoretical framework. We draw on literature from cultural evolution and the cognitive science of religion, which has -- to our knowledge -- never played a prominent role in the fantasy-reality literature. We argue this is a unique contribution in and of itself.

The reason we did not provide direct evidence for the role of ritual (among other things) is because we saw little point in attempting to establish a mechanism without first observing the proposed phenomenon. That is, we hypothesised that rituals play a special role in endorsement. If so, we should see strong endorsements for figures associated with rituals, and diminishing endorsements for those without. This we observed. Had we observed no effect, well, our hypothesis would be dead in the water and we would have wasted our time attempting to quantify a mistaken observation (that rituals are special). We do not expect our work to be the final word on the topic. Indeed, we have replicated this work (and the results) ourselves in different cultures, and subjected the topic to get a more fine-grained understanding of the proposed mechanism both observationally and experimentally (which, I do hope the reviewer will be able to read in due course!). We are fully cognizant of the limitation of this work, but I do hope the fact we replicated it gives the reviewer faith of sincere scholarly intentions. We note several passages in our text where we attempt to appropriately position our findings in the literature:

1. Page 30 (discussion): [regarding the causal factors of endorsement] *Of course, we can only make inferences to this causal factor in the present work, and we have no data to show what kind of behavioral commitments were apparent in the lives of the children we studied.*
2. And the text of the final paragraph of the discussion.

Per the reviewers alternate hypothesis: we do not suggest prominence and source play no role, indeed, we make such concessions ourselves, in several places. We have attempted to quantify this suggestion using google Ngrams, by way of simple, heuristic demonstration. Pardon our presumption, but if the author is not familiar with Google Ngrams, they might consider reading the wiki page here: <https://en.wikipedia.org/wiki/Google_Ngram_Viewer>

If the reviewer is correct, that endorsement may be predicted by prominence, then we would expect the most discussed figures to feature the most prominently in the written record, and that prominence would be roughly correlated between figures as is endorsement. Here, we present the cultural pantheon ([link](https://books.google.com/ngrams/graph?content=%22Santa+Claus%22%2C%22Tooth+Fairy%22%2C%22Easter+Bunny%22&case_insensitive=on&year_start=1990&year_end=2008&corpus=15&smoothing=3&share=&direct_url=t1%3B%2C%22%20Santa%20Claus%20%22%3B%2Cc0%3B.t4%3B%2C%22%20Tooth%20Fairy%20%22%3B%2Cc0%3B%2Cs0%3B%3B%22%20tooth%20fairy%20%22%3B%2Cc0%3B%3B%22%20Tooth%20Fairy%20%22%3B%2Cc0%3B.t4%3B%2C%22%20Easter%20Bunny%20%22%3B%2Cc0%3B%2Cs0%3B%3B%22%20Easter%20Bunny%20%22%3B%2Cc0%3B%3B%22%20Easter%20bunny%20%22%3B%2Cc0)), where we show results for ["Santa Claus","Tooth Fairy","Easter Bunny"] (case insensitive), . Note that endorsement for TF and EB is basically as high as it is for SC, and yet prominence is not. Compare and contrast the ngram results for the cultural pantheon when we also include ["Santa Claus","Tooth Fairy","Easter Bunny","dinosaur","ghost","unicorn","peter pan"] (case insensitive) ([link](https://books.google.com/ngrams/graph?content=%22Santa+Claus%22%2C%22Tooth+Fairy%22%2C%22Easter+Bunny%22%2C%22dinosaur%22%2C%22ghost%22%2C%22unicorn%22&case_insensitive=on&year_start=1990&year_end=2008&corpus=15&smoothing=3&share=&direct_url=t1%3B%2C%22%20Santa%20Claus%20%22%3B%2Cc0%3B.t4%3B%2C%22%20Tooth%20Fairy%20%22%3B%2Cc0%3B%2Cs0%3B%3B%22%20tooth%20fairy%20%22%3B%2Cc0%3B%3B%22%20Tooth%20Fairy%20%22%3B%2Cc0%3B.t4%3B%2C%22%20Easter%20Bunny%20%22%3B%2Cc0%3B%2Cs0%3B%3B%22%20Easter%20Bunny%20%22%3B%2Cc0%3B%3B%22%20Easter%20bunny%20%22%3B%2Cc0%3B.t4%3B%2C%22%20dinosaur%20%22%3B%2Cc0%3B%2Cs0%3B%3B%22%20dinosaur%20%22%3B%2Cc0%3B%3B%22%20Dinosaur%20%22%3B%2Cc0%3B.t4%3B%2C%22%20ghost%20%22%3B%2Cc0%3B%2Cs0%3B%3B%22%20ghost%20%22%3B%2Cc0%3B%3B%22%20Ghost%20%22%3B%2Cc0%3B.t4%3B%2C%22%20unicorn%20%22%3B%2Cc0%3B%2Cs0%3B%3B%22%20unicorn%20%22%3B%2Cc0%3B%3B%22%20Unicorn%20%22%3B%2Cc0)). Ghost and Dinosaur (unsurprisingly) dwarf many other categories, and yet this does not seem correlated with child endorsement. Meanwhile, Unicorn is roughly as prominent as santa, yet endorsed far below him, and well above the other cultural figures. Indeed, Peter pan is far more prominent than the Tooth Fairy. All this is to say, that while other explanations exist (which we do not immediately discount at all; indeed, please note we took them very seriously in this review), they cannot immediately replace the existing hypotheses based on this rather rough-and-ready data. We hope our data can be considered as the first step in theory building, in which we demonstrate a proof-of-concept for the special role ritual plays in fantasy-reality distinction, and that our humble attempt at theory building be judged by those who consume the literature.

4. I am sympathetic to the authors' proposal that the rituals and norms associated with certain figures might constitute a form of testimony. However, I doubt whether a non-experimental study in which figures that are presumed to be associated with rituals and norms are compared with figures that are presumed to not be so associated are compared can provide strong evidence for their claim. Those two classes of figure will be very likely to vary in other ways (see comment #3 above), weakening any conclusions that can be drawn.

We hope to have addressed this question in response to the last critique: our goal was to expand an existing literature by drawing in a new body of work, and to demonstrate a proof of concept. We hope our attempt at theory building can be regarded as a first-step (one bolstered by an immediate and transparent replication) which can serve future scholars. The only conclusion we hope readers draw is that they should take the role of rituals as a form of testimony seriously, and that a pantheon may be a theoretically valuable frame-work. Indeed, even without amendment, our final paragraph emphasises these points (emphasis added):

*“The data we present here suggests that, when children (and even adults) are asked to report their subjective confidence that various kinds of real and non-real figures exists, they are not only able to do so, but do so with nuance, and in a consistent pattern apparent at the population level.* ***We argue that the possibility that cultural rituals and normative requirements on the part of figures like Santa, the Easter Bunny, and the Tooth Fairy may be very powerful cultural tools that lead to children believing such figures are real. And we anticipate - but cannot address directly - that ritual involvement is a key determinant in adult beliefs for more institutional supernatural figures such as the deities of major world religions.*** *We hope that treating the corpus of real, supernatural, and fictional figures as a kind of pantheon, united by a coherent structure of underlying qualities,* ***opens the door for higher resolution understanding of how children come to understand what is real and what is not (even when they are wrong), and allow for more nuanced approaches to research when investigating the predictors of belief and endorsement****. In addition to testimony, content, and source information,* ***we must pay more attention to the specific role that cultural rituals play in widespread belief of culture-bound supernatural figures.”***

Reviewer #2: This paper presents the results of two surveys of children’s and adults’ beliefs about a range of real and fantastical beings. Specifically, the authors presented participants with various beings that they had categorized a priori into 5 groups as a function of a number of characteristics and attributes that they reasoned would affect judgments about reality. Participants were asked to indicate how real they thought the entities were, using a Likert scale. Results indicate that children and adults exhibit different patterns of judgments regarding these entities, and are interpreted as revealing that children’s judgments reflect the predicted characteristics/attributes of the beings, and specifically the important role of participation in the behavioral rituals surrounding the entities.

This study is unique in attempting to explore a more nuanced representation of reality than in previous studies of this topic, many of which have simply assessed beliefs about whether something is real or not real. This is a laudable goal. The focus on the role of involvement in behavioral rituals is also novel and important. In general, I really like that the authors are trying to determine how various factors like ritual participants and evidence interact to engender belief.

I should note that I reviewed an earlier version of this paper for another journal. I mention this, because I find this version significantly improved over the original version, and that affects my evaluation (in a positive way). Overall, though, I still have some concerns about the paper, and they follow.

1. On p. 20, the authors discuss whether endorsement becomes more adult-like with age. The text states that, “it is clear from the data presented in Figure 4 that the negative slopes associated with fictional characters and cultural characters..” From the graph that appears to me to be Figure 4, it doesn’t look like there’s any decline at all in belief in cultural figures—that line looks flat. First, this doesn’t fit with the text, and second, there’s so much research showing that these beliefs decline with age that something must be odd about these data. Can the authors clarify?

What we initially believed is happening here (in both Study 1 and Study 2) is that the relationship is actually non-linear, specifically an S-shaped curve, where across the given range of ages (3 - 11; and 4-12 respectively) there is a rather precipitous decline in endorsement for Santa at around the ages of 7 or 8. Variance in our samples, coupled with the fact that study 2 (which has the steeper linear curve) has a lower bottom value and a higher top value is consistent with this. The other possibility is that in study 1 -- when children are reporting results to their parents -- that they are deceiving their parents so as to maintain their [parent’s] belief that they [the child] still believe in Santa. Both of these explanations, in tandem, may reflect that value. In study 2 the value is noticeably steeper, likely consistent with a reduced drive for deception. We have included a discussion about this specific point (in study 1) in the discussion of study 1.

However, what appears to be happening is partially an issue with sampling. When we plot the data from both studies on Age vs. Santa_endorsement we get the scatter plot (see figure 1; In study 1, 6 children over the age of 7 did not report a value for this measure; 20 children under this age did not report a value; In study 2, 2 missing values in children over 7, 7 missing values for children over 7).

While there are fewer datapoints *generally* on santa endorsement above the age of 7, there are *proportionally* more values that are not at ceiling. We have no compelling evidence here for a non-linear relationship, but it does appear to explain the nature of the regression lines - the model is more influenced by younger values than older ones.

And in these figures, we included Santa (sreal), tooth fairy (treal), and easter bunny (ereal) in the graphs. Again, there are fewer children above the age of 7 than below, but it is clear that there are proportionally more middling-responses above that age.

Per the comment about consistency fitting with the text. We have amended the section in Study 1 under the heading ‘Hypothesis 4: Does age predict a more adult-like understanding of figures’, to include the following:

*Notably, the slope for Cultural Figures, while nominally negative, is largely flat. We have two possible explanations for this: the first is that children who do not believe in these cultural figures, when reporting to their parents, are partially motivated to deceive their parents that they actually do. The second is that belief in cultural figures is likely an S-shaped curve, where belief declines precipitously around the ages of 7 or 8 (Anderson and Prentice 1994), and that having fit a linear model, we are unable to capture the true nature of this effect. Study 2 addresses the former concern. We also note that our data contains proportionally fewer children over the age of 7 than it does under. Unfortunately, only future research will address this.*

2. As the authors note, in terms of the hierarchy, for the most part the data from Study 2 replicate Study 1. But I was struck by the differences in the correlations. **For the most part, they’re lower in Study 2**. Could the authors speculate on why this might be the case?

We thank the reviewer for bringing this up. We hadn’t actually made this specific observation, having generally focussed on the direction, and whether or not the values met the assumptions of the cluster analysis. This is fascinating, and we do not have a ready explanation, save for the obvious: the data collection method was different. It’s not obvious, however, why the correlations would be systematically diminished as a result of this. My colleagues and I have discussed this, and no obvious explanation presents itself, outside the obvious methodological difference, but we are at a lost to contextualise or explain it. We would welcome further insight, if appropriate. Should the reviewer wish that these comments were included in print, we can oblige.

3. Another difference between the two studies is that in Study 2, the slope for Cultural Figures actually does appear to be negative. Why this difference? The authors make much in the General Discussion regarding how the Study 2 results validate the method used in Study 1 but this is a critical difference. Apparently in an attempt to explain it, they state that it is probably due to belief declining abruptly rather than gradually (this is stated both in the discussion of Study 2 and in the General Discussion). First, I don’t understand how that explains the difference. Could the authors expand upon their explanation? Second, I only know of one study that looks specifically at Santa disbelief, and that study finds that belief does not decline abruptly: As Anderson & Prentice (1994) state, “the ultimate realization came about through a gradual transition to disbelief rather than at a single point of demarcation” (p. 74).

Regarding Anderson and Prentice: This data is consistent with our own, broadly put. Noting that A&P do not provide empirical evidence of endorsement/belief, but rather report how often children ‘had doubts’ (which is, of course, very useful). Many children (ages 9 - 12 in their sample) reported doubts, but not the duration they held them. Accepting that this is a true claim, the scale at which these doubts are gradual is important. Were they gradual over the course of 1 year? Or over the course of 8 years? Our study looks at cross-sectional data from 3 to 11 years. When we suggest a precipitous drop, we do not mean that on a given tuesday afternoon they were struck with a realization, but simply that disbelief could emerge in the 7th or 8th year, and not be accurately reflected by a linear model over the 8 year span we examine. Moreover, that children can ‘sort of’ believe in santa supports our decision to allow for a graded scale.

In a previous question we indicated that we discussed the issue of sampling in the over-7-year bracket, and we refer again to that section to addressed the concerns expressed by the reviewer.

4. On p. 28, the authors state that “endorsement for cultural, ambiguous, and fictional figures declines at differential rates” but it looks to me like in Study 2 (Figure 9, I think), the lines for cultural and ambiguous are parallel. (And, as already mentioned, in Study 1 it looks like cultural figures doesn’t decline). Could the authors clarify?

Regression coefficients in a MLM cannot be independently interpreted as equivalent to regression coefficients in a series of separate regressions. One might conduct 5 linear regression, predicting endorsement for each of the five categories, and might then compare the standardized values and y-intercepts. This would be intuitively appealing, but would ignore the covariance between the categories that can be handled by a single MLM with five variables. While regression coefficients can be extracted, one must examine the model fit. In our case, the model is a better fit when we allow the y-intercepts to vary *and* when we allow the slopes to vary. To the best of our knowledge we cannot constrain some slopes and not others (while still maintaining the integrity of the proposed categories). And so, our statement that the ‘decline at differential rates’ is accurate in the sense that ‘freely estimating the slopes of each categories is superior to constraining the slopes to a single regression coefficient’. We understand how we have been loose with our language here, and have deleted the offending sentence, and included a footnote (page 28) clarifying the nature of the analysis/results/interpretation

5. I’m a little confused about the paragraph at the bottom of p. 28 as well. The text states that dinosaurs aggregated with The Wiggles. But on p. 24 they state that Dinosaurs and Wiggles didn’t aggregate (and that in the “primary dataset this cluster had relatively low reliability”). Please clarify.

We have amended the offending sentence: *But rather, dinosaurs aggregated with The Wiggles in study 1 and that dinosaurs and wiggles did not aggregate in study 2*

6. On p. 30 the authors state that the “most unbelievable figures” assume the lowest rank in the pantheon. What is meant by “most unbelievable figures”? They also state that ambiguous figures “assume a higher standing than that of cultural figures.” I guess I’d like the language to be more precise in both of these cases. Also, it doesn’t actually seem to be true. In Study 2, it looks like fictitious and ambiguous features are changing their relative standing and in Study 1 there is no change in standing (again, I’m looking at what I think is Figure 9).

We have amended the sentence to read more precisely, moreover, the confusion that the reviewer pointed out in figure 9 was a error on our part. We meant ‘virtually real’ not ‘ambiguous’. We appreciate the reviewer noting this error:*the figures which we predicted to be least endorsed assume the lowest rank in the pantheon, while it is in this period that ‘virtually real’ figures* (such as dinosaurs and humans appearing on television) *appear to assume a higher standing than that of cultural figures (primarily observable in study 2).*

7. On p. 33 the authors seem to be discussing individual subject patterns but I didn’t see any discussion or analysis of these in the Results sections. The authors should include these data if they want to discuss them.

We have amended that sentence to read: *While regrettable, we note that the same problem does not occur for the ambiguous and cultural figures, which tend to reveal middling endorsements by individuals (in the former case; as revealed by the scatter plots of individual values in figure 7), and high confidence in their existence in the latter.*

8. There are numerous grammatical errors through the manuscript. I recommend a very close checking before submitting a revision.

We hope to have caught these errors. Thank you.

Some minor points:

1. P. 10, states that “endorsement between these figures is not statistically different.” First, the authors needs to specific to which figures they are referring, that is, between Santa and the other two, or between the other two? Second, a citation needs to be provided for this claim.

We have amended the line to read: It is an empirical question whether or not this makes a difference in endorsement for Santa relative to the Easter Bunny or the Tooth Fairy - and it appears that endorsement between these figures is not statistically different (as revealed by our own data).

2. P. 10, what is meant by the “four factors of testimony”?

We have amended the relevant section: While not perfectly equivalent, in the context of the four factors of testimony (direct- and indirect evidence, rituals/norms, and fictional disclaimers) that lead to endorsement,

3. For Study 1, 59 out of 154 children’s data were dropped. Were these all due to not answering the last question? I’d like a little more information about this and its implications regarding the generalizability of the findings.

We indicated that using the final question was our flag, and that “among those that failed to answer the final question, none responded to more than 3 figures”. That is, of those, who didn’t answer the final question, they completed less than half of all questions.

We don’t know why participants in this study dropped out half-way through, perhaps the child lost interest? Perhaps the parents grew uncomfortable with the questions if their child became increasingly skeptical. It’s unclear in what manner this influenced the results. We hope that our replication in study 2, largely demonstrated the same data, suggests that whatever effect the missing children had, it was not producing a reliably effect on the data.

4. Pp. 14-20, the “hypotheses” in the subheadings aren’t hypotheses. I’d suggest rephrasing.

We are unsure in what we can change our hypothesis to conform to this request. Perhaps an example would be of use? We note that the reviewer was not confused by our predictions or analysis, and so are unsure in what way re-phrasing will clarify for other readers.

5. P. 20 second paragraph, author should indicate explicitly that these data concern the “Ambiguous” category members.

We have amended this sentence.

6. None of the (main) figures in my copy are labeled so I had to guess figure numbers.

We apologise for this.

7. What’s wrong with including 3-year-olds (p. 33)? Lots of studies on this topic do so.

We agree. Previous reviewers thought including 3 year old was an issue in analysis and interpretation. Thus we have been quite explicit in our defence of their inclusion (though we generally believe we need not be).
